# Repeated Interval Loughborough Soccer Passing Tests: An Ecologically Valid Motor Task to Induce Mental Fatigue in Soccer

**DOI:** 10.3389/fphys.2021.803528

**Published:** 2022-01-20

**Authors:** Chao Bian, Ajmol Ali, George P. Nassis, Yongming Li

**Affiliations:** ^1^School of Physical Education and Training, Shanghai University of Sport, Shanghai, China; ^2^School of Sport, Exercise and Nutrition, Massey University, Auckland, New Zealand; ^3^Department of Sports Science and Physical Education, The Chinese University of Hong Kong, Shatin, China; ^4^Physical Education Department, College of Education (CEDU), United Arab Emirates University, Al Ain, United Arab Emirates; ^5^Department of Sports Science and Clinical Biomechanics, SDU Sport and Health Sciences Cluster (SHSC), Faculty of Health Sciences, University of Southern Denmark, Odense, Denmark; ^6^China Institute of Sport Science, Beijing, China

**Keywords:** inducement, LSPT, soccer-specific, cognitive, skill

## Abstract

Most studies investigating mental fatigue (MF) in soccer utilized a computerized Stroop task to induce MF. However, the traditional key-pressing task has been challenged for its lack of ecological validity. The limited relevance to real-life soccer made it difficult to bridge the gap between the research and the applied setting. Therefore, a novel soccer-specific inducing task is in urgent need. This study compared a novel MF-inducing task in soccer with the Stroop task and investigated the impact of induced MF on cognitive and soccer-specific skill performance. A randomized, counterbalanced crossover design was employed. Fifteen well-trained male soccer players randomly participated in three MF-inducing tasks. Two of them were motor tasks consisting of 10 repeated interval Loughborough Soccer Passing Test (10xLSPT or LSPT) in clockwise passing order (10xC-LSPT) with each block starting every 2 min. The two tasks share the same movement pattern, but C-LSPT is considered to have lower cognitive demands. The third was the 20-min Stroop task (Stroop-20). MF was assessed immediately before and after each task by visual analog scale (VAS), the cognitive performance in a 3-min Stroop task, and the skill performance in one LSPT. Subjective MF increased similarly after 10xLSPT and Stroop-20 (+ 25.4 ± 10.3 vs. + 23.4 ± 10.8 AU, *p* = 0.607). The induced MF by 10xLSPT and Stroop-20 had no impact on cognitive performance and movement time but similarly affected in a significantly negative manner on penalty time (+ 5.9 ± 4.9 vs. + 5.4 ± 4.2 s, *p* = 0.748) and passing accuracy (–1.4 ± 1.5 vs. –1.0 ± 1.3, *p* = 0.465). Two motor tasks shared similar intensity, but 10xC-LSPT was inefficient to induce MF. The results showed that the 20-min repeated interval LSPT could induce a similar MF as the Stroop task. The induced MF had detrimental effects on soccer skill performance. The novel motor task is recommended for MF studies in soccer as an inducement task. Practitioners should be cautious about the prolonged cognitive-demanding skill section of the pre-match warm-up to avoid the negative effect of MF on the upcoming match. This motor task pattern could be followed as a supplementary training protocol.

## Introduction

Soccer is an open-skill sport of high unpredictability, requiring players to have extraordinary physiological capacities combined with outstanding abilities in the areas of motor control, perception, and cognitive functioning (Romeas et al., 2016; Hans-Erik and Daniel, 2019). In matches, players have to maintain high attention levels over prolonged periods, perceive and interpret relevant information correctly, and then select the appropriate motor response under the constraints of time and space pressure (Baker et al., 2003; Nédélec et al., 2012). These soccer-specific tasks will cause mental fatigue (MF) in players (Coutts, 2016), a psychobiological state characterized by the feelings of tiredness and lack of energy during/after long periods of cognitive activity (Boksem and Tops, 2008; Marcora et al., 2009). Recently, there has been a growing interest in MF on soccer performance with negative impacts on physical (Smith et al., 2015, 2016a; Coutinho et al., 2018; Filipas et al., 2020; Trecroci et al., 2020), technical (Badin et al., 2016; Smith et al., 2016a,^2017^; Greco et al., 2017; Filipas et al., 2020; Trecroci et al., 2020), tactical (Coutinho et al., 2017, 2018; Kunrath et al., 2018, 2020b), and cognitive (Smith et al., 2016b; Fortes et al., 2019, 2020; Gantois et al., 2020; Trecroci et al., 2020) performance of soccer players.

Most studies investigating MF in soccer utilized a computerized cognitive task (i.e., Stroop task) to induce MF (Kunrath et al., 2020a). Despite the effectiveness in inducing MF, traditional key-pressing tasks such as the Stroop task have been challenged recently due to its lack of ecological validity in this specific research field (Smith et al., 2018; Thompson et al., 2019). The limited relevance to real-life soccer made it difficult to bridge the gap between the research and the applied setting. Therefore, a novel motor task with ecological validity for soccer is in urgent need (Pinder et al., 2011; Travassos et al., 2012). Coutinho et al. (2017) have used a 20-min whole-body coordination task to induce MF. This motor task involved performing 7 different step exercises with a ladder while juggling a tennis ball to increase attentional and cognitive demands (Coutinho et al., 2017). Despite the success in inducing MF, this task might not be soccer-specific.

The Loughborough Soccer Passing Test (LSPT) is a multifaceted soccer-specific skill test to evaluate passing, dribbling, controlling, and decision-making abilities and has been shown to be a valid and reliable indicator of soccer skill performance (Ali et al., 2007). In this test, players are required to react to verbal random passing orders 16 times and execute skills quickly and accurately under time and space pressure (Ali et al., 2007). The finishing time (movement time) of one LSPT is less than 1 min. Relative intense cognitive and skill demands may cause a state of MF; if this happens, LSPT repeatedly performed for 20 min may be considered an effective ecological task for inducing MF in soccer players.

Therefore, the aim of this study was to explore a novel motor task for inducing MF in soccer with a better ecological validity by comparing the MF induced by repeated interval LSPT and the Stroop task. Secondarily, this study aimed to investigate the impact of induced MF on cognitive and soccer skill performance. Previous findings show that a 20-min motor task can induce the perception of MF of players to similar levels as responses to the Stroop task (Coutinho et al., 2017). Therefore, we hypothesized that 10-time repeated interval LSPT (20 min) would induce the same degree of MF as the 20-min Stroop task, and induced MF would negatively affect cognitive and soccer skill performance.

## Materials and Methods

### Participants

The total sample size calculated by G*Power for a moderate effect size of 0.5 for performance indicators, an α of 0.05, and a power of 0.8 (1–β) was 42, with a minimum of 14 subjects in each group (3 sessions) in the counterbalanced crossover design. To account for any potential dropout, we recruited 15 well-trained male soccer players (age = 22.0 ± 2.5 years, height = 174.5 ± 6.5 cm, body mass = 68.2 ± 7.6 kg, body fat = 14.5 ± 2.9%, training experience = 8.1 ± 2.7 years, weekly training duration = 7.2 ± 1.0 h), including 5 forward, 5 midfielders, and 5 defenders. Participants were free of any known disease/injury/sleep disorder/smoking/medication. They were informed to sleep for at least 8 h the night before the testing sessions to avoid any cognitively demanding activity the day before the tests and to refrain from alcohol for 24 h and caffeine for at least 12 h before the tests (Craig et al., 2012). Players were instructed to consume water and the same light meal 1.5 h before every testing session. An information sheet was provided to the head coach and players at the beginning to explain the procedures involved, and they were informed that they could withdraw from the study at any time. Participants signed informed consent forms prior to commencing the study, and all procedures were approved by the Human Research Ethics Committee of the Shanghai University of Sport.

### Study Design

**FIGURE 1:**
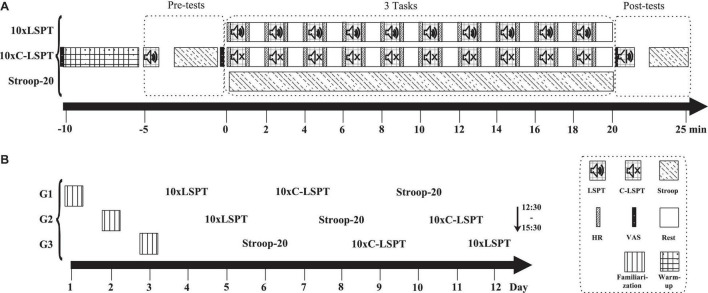
Timelines of study procedures. **(A)** Timeline of test sessions; **(B)** timeline of study design; LSPT, Loughborough Soccer Passing Test; 10xLSPT, 10 repeats of Loughborough Soccer Passing Test; C-LSPT, modified Loughborough Soccer Passing Test in a clockwise passing order; 10xC-LSPT, 10 repeats of modified Loughborough Soccer Passing Test in a clockwise passing order; Stroop, Stroop task; Stroop-20, 20-min Stroop task; VAS, visual analog scale; HR, heart rate; G, group of participants.

A randomized, counterbalanced crossover design was employed. The purpose of this study was masked by telling players to compete with teammates for best performance. Participants visited an indoor parquet court with indoor soccer boots four times, with each trial separated by 48 h. The testing procedure is presented in Figure 1. The first visit was used for familiarization, with the following three visits as testing sessions. As illustrated in Figure 1A, all the sessions were comprised of (1) a warm-up, (2) pretests, (3) 20-min MF-inducing task, and (4) posttests. The tasks utilized to induce MF in session 1 and session 2 were repeated interval LSPT, with either a randomized order of passing [i.e., 10 repeated interval Loughborough Soccer Passing Test (10xLSPT)] or clockwise order of passing (10xC-LSPT), while the task in session 3 was the Stroop task (Stroop-20). After familiarization, players were divided into three groups randomly (denote: G1/2/3). Five players in each group conducted one session at a fixed day time (12:30–15:30) to minimize any potential effect of circadian rhythm (Figure 1B).

### Procedures

During familiarization, players were exposed to all tasks and procedures together with the clear definition of MF. Familiarization included performing at least 5 attempts of the LSPT under the instruction of examiners, 3 attempts of 3-min Stroop task (Stroop-3), and 3 attempts of sliding visual analog scale (VAS) until players were comfortable with the test procedures and their scores indicated that the learning effect had diminished (i.e., a plateau in scores) (Foskett et al., 2009; Smith et al., 2019).

The warm-up lasted 5 min and included ball passing and dynamic stretching. Players then finished one LSPT for assessing the initial soccer-specific skill performance and rested for 1 min, followed by a Stroop-3 for initial cognitive performance. After each MF-inducing task, one LSPT and Stroop-3 were conducted again for posttests. Players completed the VAS prior to the warm-up, immediately before and after the MF-inducing tasks. The first VAS score was used to assess the motivation of players for the upcoming session. The second and third VAS scores were used to quantify the baseline MF and the MF perceived after tasks.

### Mental Fatigue Assessment Indicators

The assessments of MF in this study can be categorized as a subjective report, cognitive performance, and soccer-specific skill performance.

The changes of VAS values immediately before and after the MF-inducing tasks indicated the subjective perception of MF. The Stroop-3 in the pre- and posttest was used to assess the cognitive performance with the measures of response time and response accuracy. The LSPT in the pre- and posttest was used to evaluate soccer skill performance with the measures of movement time, penalty time, and passing accuracy (number of perfect passes).

### Visual Analog Scale

The scale was plotted on a customized plastic ruler of 100 mm and anchored by the extreme limits of “minimum” (0) and “maximum” (100). One side was printed with a single straight line for participants while the other side was printed with the tick mark and number for examiners. Players were instructed to indicate “*What do you think of your perception of mental fatigue at this moment?*” by sliding an attached transparent vernier along the ruler from left to right until they were satisfied with the location. A subjective score (AU) was obtained by reading the millimeter distance from the left side of the scale to the vernier indicated by the player.

During familiarization and before every session, players were provided with the following definition of MF to support their subjective assessment: “*Mental fatigue, different from physical fatigue, is a psychobiological state characterized by feelings of tiredness and a lack of energy and is induced by prolonged periods of demanding cognitive activity*” (Boksem and Tops, 2008; Marcora et al., 2009; Smith et al., 2016b) (if confused, the examples of examination or studying for an extended period were given).

Motivation for upcoming sessions was also measured by the 100-mm VAS as reported by previous research (Smith et al., 2016b). Participants were asked “*How motivated are you to want to accomplish this test?*” before each session.

### Stroop Task

The Stroop task in the pre- and posttest was the version reported by Badin et al. (2016) and Gantois et al. (2020). The MF-inducing task in session 3 was a continuous Stroop task lasting 20 min (Stroop-20). Players were instructed to sit on the bench at the corner of the court and to hold the tablet (iPad, Apple Inc., California, United States) to perform the Stroop task individually under the supervision of one examiner. Players were provided noise-canceling headphones to minimize distractions. Gestures were only given for indicating the start and end of the task. Stroop tasks were programmed by E-Prime (Psychology Software Tools, PA, United States) and carried out on a full-HD screen (2,732 × 2,048 pixels, 12.9 inch) tablet; the virtual keyboard of the tablet was used for key pressing.

Colored words (red, blue, green, and yellow) were presented one at a time on the screen with a black background. Trials were arranged in a pseudorandom order with 50% of them being congruent (matched word and color) and 50% being incongruent, with all incongruent word–color combinations occurring with equal frequency. Players were required to respond to each trial by pressing one of the four keys on the bottom edge of the screen and then to choose the color of the word rather than its meaning. However, to increase task difficulty, if the word was displayed on screen in the color of red, the correct response was to press the key corresponding to the meaning of the word (Badin et al., 2016). The stimulus did not fade from the screen until a response was given. When the answer was correct, the stimulus disappeared, and a new one was set immediately, while any incorrect answer elicited a beep sound to prompt more accurate performance and a new stimulus subsequently appeared (Gantois et al., 2020). Moreover, to prevent the subjective slack during the task, we established the cutoff value of mean response time at 1.5 s, any slower data (<800 trials) were excluded for analysis. Players were motivated to perform faster than the cutoff value by the provision of an additional ¥50 RMB reward.

In the pre- and posttest, the 3-min short version of the Stroop task (Stroop-3) was used to assess the cognitive performance, with the measures of response time and response accuracy. Stroop-3 followed all the rules as Stroop-20, during which the player was asked to respond no less than 120 trials.

### The Loughborough Soccer Passing Test

**FIGURE 2:**
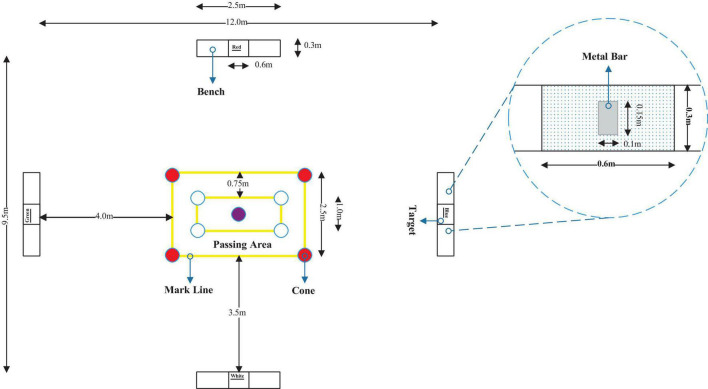
Schematic representation of the Loughborough Soccer Passing Test (LSPT).

Players stood in the center of a rectangular passing area (2.5 × 4 m, Figure 2) and responded to an audible signal which indicated the direction of the pass. The four colored passing targets (0.6 × 0.3 m) were placed on rebound boards (benches), surrounding the rectangular playing area (12 × 9.5 m). Participants were required to complete the passes as quickly as possible while minimizing errors. Penalty time was added for errors (e.g., passing inaccuracy, playing from an incorrect zone, and poor ball control). Penalty time was deducted for a “perfect” pass (hitting the metal bar attached to the middle of the target), while movement time was the time to complete the test. One examiner was involved in calling out the order and timing the test. The second examiner was in charge of judging the errors and scoring the performance (refer to Ali et al., 2007 for details).

One standard LSPT involved 16 passes, using a randomized order of pass direction, as used in the pre- and posttests. The LSPT that was utilized to induce MF was either repeated interval LSPT (i.e., 10xLSPT) or repeated interval modified LSPT with clockwise order of passing (i.e., 10xC-LSPT). Since the finishing time of one LSPT was approximately 45 s, the 20-min MF-inducing process involved 10 repeats of the LSPT or C-LSPT, with each starting every 2 min. During the intervals between each LSPT and C-LSPT, the player rested for approximately 75 s in a standing posture.

### Intensity of Two Motor Tasks

Heart rate (HR) was monitored throughout utilizing an HR chest strap (Polar H10, Kempele, Finland) (sampling at a frequency of 2.4 GHz). HR immediately before and after each LSPT or C-LSPT during session 1 and session 2 was recorded for analyzing the physical activity (intensity) during the soccer-specific motor tasks. The Stroop task in session 3 was a static key-pressing task, the average HR of players was at their resting level according to the preexperiment, and therefore, this set of HR data was excluded for the analysis.

### Statistical Analysis

All data are presented as mean ± SD unless otherwise stated. All statistical analyses were completed using SPSS Statistics, version 25 (IBM, NY, United States). Data were tested for normality and log-transformed when necessary. The motivation for the upcoming sessions was analyzed by a one-way ANOVA. Two-way repeated-measures ANOVA was performed to identify differences in HR, cognitive, and soccer skill indicators according to time and testing sessions (treatment) comparisons. Data sphericity was verified by Mauchly’s test, and the Greenhouse-Geisser correction was adopted when this assumption was violated. Pairwise comparisons were assessed using the Bonferroni correction. The pre- and post-VAS scores were compared by paired *t*-tests. As a subjective indicator, players report different pre-VAS scores and have different feelings of MF every day. Therefore, we only considered the Time variable (intragroup) without the Treatment variable (intergroup) for VAS scores. The comparisons within sessions (time), as well as the comparisons between sessions (treatment), were also reported using mean difference ± SD. Posttest value minus pretest value: positive values meant an increase while negative values referred to a decrease over time. Significance was set at 0.05 (2-tailed) for all analyses. Partial eta squared effect sizes (η^2^*_*p*_*) were reported (< 0.04, no effect; 0.04–0.24, minimum practical effect; 0.25–0.63, moderate effect; ≥ 0.64, strong effect) (Ferguson, 2016).

## Results

### Subjective Mental Fatigue and Motivation

**FIGURE 3:**
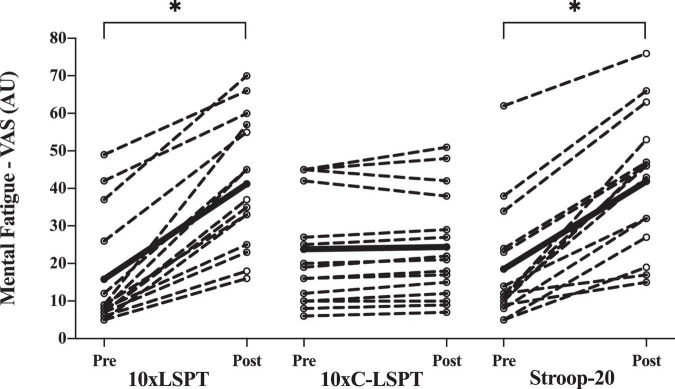
Pre-post assessment of subjective mental fatigue. Circles and dashed lines indicate the data of individual participants, and black solid lines indicate the mean value. *Significant changes between pre- and posttest (*p* < 0.05); VAS, visual analog scale; 10xLSPT, repeated interval Loughborough Soccer Passing Test; 10xC-LSPT, repeated interval modified Loughborough Soccer Passing Test in clockwise order of pass direction; Stroop-20, 20-min Stroop task.

Initial subjective MF that was assessed by VAS before the three sessions was similar (15.8 ± 15.0 vs. 23.8 ± 15.5 vs. 18.5 ± 15.6 AU, respectively; *p* = 0.359). There were similar significant increases in subjective MF (Figure 3) after 10xLSPT and Stroop-20 (+ 25.4 ± 10.3 vs. + 23.4 ± 10.8 AU; *p* = 0.607), but no significant change after 10xC-LSPT (*p* = 0.913). Players had similar motivation among the three sessions (87.8 ± 7.6 vs. 87.3 ± 7.0 vs. 86.9 ± 6.6 AU, respectively; *p* = 0.937).

### Intensity of Two Motor Tasks

**FIGURE 4:**
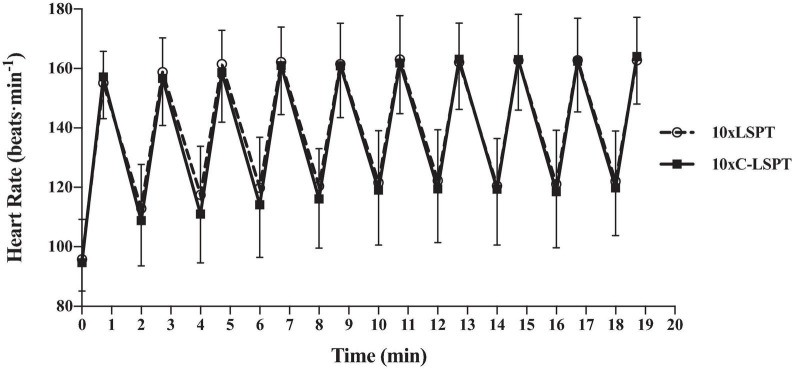
Heart rate in two motor tasks. 10xLSPT, 10 repeats of Loughborough Soccer Passing Test; 10xC-LSPT, 10 repeats of modified Loughborough Soccer Passing Test in clockwise order of pass direction.

There was no difference in mean HR throughout 10xLSPT (139 ± 14 bpm) and 10xC-LSPT (138 ± 14 bpm; *p* = 0.731; Figure 4).

### Cognitive Performance

**FIGURE 5:**
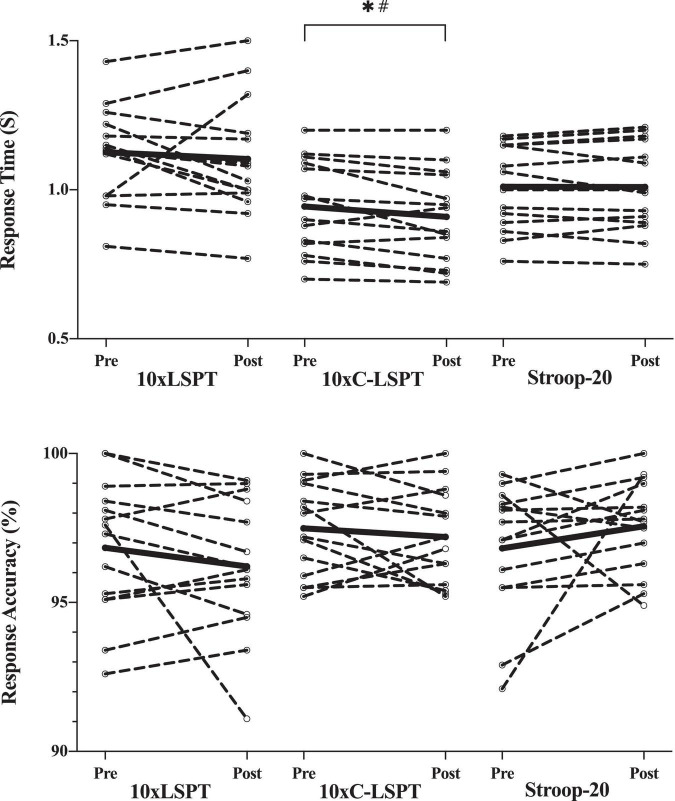
Cognitive performance in pre- and posttests. The response time data of one player were excluded (> 1.5 s). Circles and dashed lines indicate the data of individual participants, and black solid lines indicate the mean value. *Significant changes when compared with pretest (*p* < 0.05); ^#^moderate effect; 10xLSPT, repeated interval Loughborough Soccer Passing Test; 10xC-LSPT, repeated interval modified Loughborough Soccer Passing Test in clockwise order of pass direction; Stroop-20, 20-min Stroop task.

As illustrated in Figure 5, there was no main effect of time [*F*(1, 39) = 2.113; *p* = 0.154, η^2^*_*p*_* = 0.051] or treatment-time interaction [*F*(2, 39) = 0.543; *p* = 0.585, η^2^*_*p*_* = 0.027] in the response time of the Stroop-3 in the pre- and posttests of all three sessions. However, there was a main effect of treatment [*F*(2, 39) = 5.113; *p* = 0.011, η^2^*_*p*_* = 0.208] in the response time for Stroop-3, with lowest response time in 10xC-LSPT (0.93 ± 0.15 s; *p* < 0.05) relative to other two trials, but no differences between 10xLSPT and Stroop-20 (*p* = 0.249). *Post hoc* analysis showed similar steady trend over time in 10xLSPT (*p* = 0.733) and Stroop-20 (*p* = 0.990), while there was a significant decrease in 10xC-LSPT (–0.034 ± 0.05 s; *p* = 0.023, η^2^*_*p*_* = 0.337).

When analyzing the response accuracy of Stroop-3, no significant main effect of time [*F*(1, 13) = 0.030; *p* = 0.866, η^2^*_*p*_* = 0.002], treatment [*F*(2, 26) = 1.480; *p* = 0.246, η^2^*_*p*_* = 0.102], and treatment-time interaction [*F*(2, 26) = 2.256; *p* = 0.144, η^2^*_*p*_* = 0.148] was found. The pre-post accuracy remained unchanged, and the mean accuracy was similar (0.97 ± 0.02) in three sessions.

### Soccer Skill Performance

**FIGURE 6:**
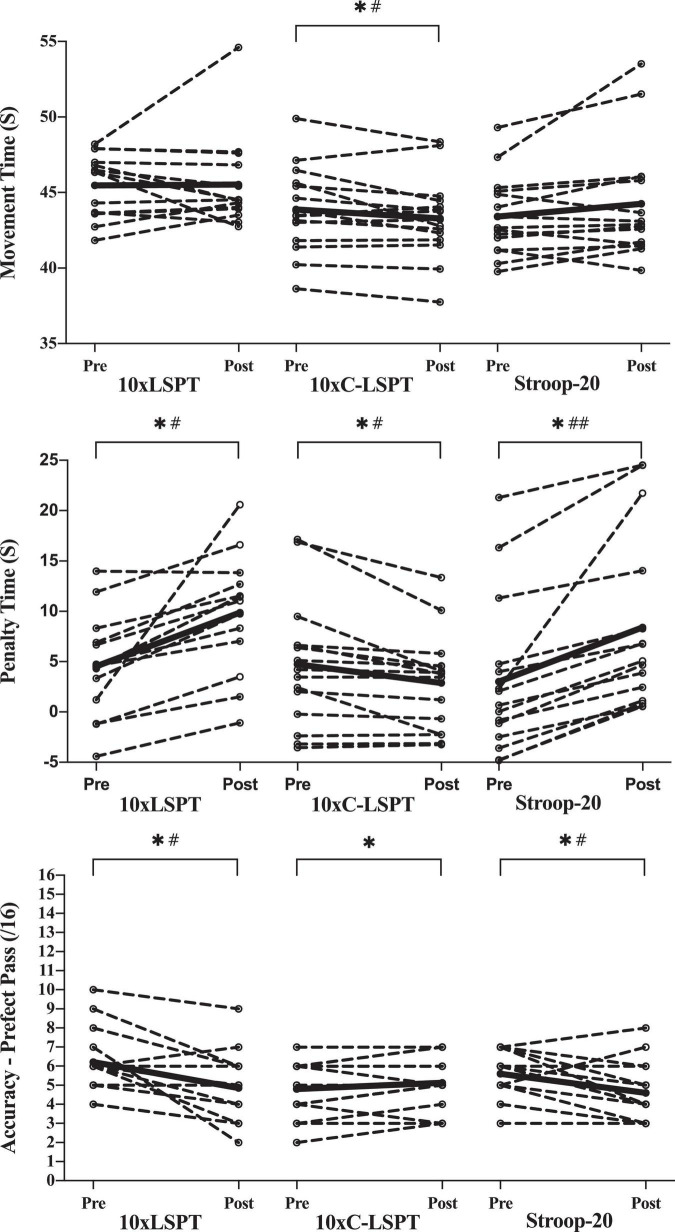
Assessment of soccer skill performance. Circles and dashed lines indicate the data of individual participants, and black solid lines indicate the mean value. *Significant changes when compared with pretest (*p* < 0.05); ^#^moderate effect; ^##^strong effect; the response time data of one player were excluded (>1.5 s); 10xLSPT, repeated interval Loughborough Soccer Passing Test; 10xC-LSPT, repeated interval modified Loughborough Soccer Passing Test in clockwise order of pass direction; Stroop-20, 20-min Stroop task.

As presented in Figure 6, the movement time of LSPT indicated no significant changes after two MF inducement tasks of 10xLSPT (*p* = 0.983) and Stroop-20 (*p* = 0.098). However, there was a significant decrease in movement time after 10xC-LSPT (–0.587 ± 1.040 s; *p* = 0.046; η^2^*_*p*_* = 0.254).

For penalty time of LSPT, significant main effect of time [*F*(1, 42) = 28.462; *p* < 0.001, η^2^*_*p*_* = 0.404] and treatment-time interaction [*F*(2, 42) = 18.038; *p* < 0.001, η^2^*_*p*_* = 0.462], but no significant main effect of treatment [*F*(2, 42) = 1.519; *p* = 0.231, η^2^*_*p*_* = 0.067] was found. There were similar significant increases in penalty time after 10xLSPT (+ 5.921 ± 4.923 s; *p* < 0.001; η^2^*_*p*_* = 0.608) and Stroop-20 (+ 5.373 ± 4.168 s; *p* < 0.001; η^2^*_*p*_* = 0.640). However, 10xC-LSPT caused a significant reduction in penalty time (–1.853 ± 2.314 s; *p* = 0.008; η^2^*_*p*_* = 0.407).

There existed a significant main effect of time [*F*(1, 42) = 14.252; *p* < 0.001, η^2^*_*p*_* = 0.253] and treatment-time interaction [*F*(2, 42) = 8.246; *p* = 0.001, η^2^*_*p*_* = 0.282], but no significant main effect of treatment [*F*(2, 42) = 1.071; *p* = 0.352, η^2^*_*p*_* = 0.049] for the amount of perfect passes (passing accuracy) of LSPT. The passing accuracy significantly decreased after 10xLSPT (–1.4 ± 1.502; *p* = 0.003; η^2^*_*p*_* = 0.482) and Stroop-20 (–1.000 ± 1.309; *p* = 0.01; η^2^*_*p*_* = 0.385), while there was no significant change after 10xC-LSPT (*p* = 0.096; η^2^*_*p*_* = 0.185).

## Discussion

The primary purpose of this study was to explore a novel motor task for inducing MF in soccer by comparing the MF induced by 20-min repeated interval LSPT and computerized Stroop task. The 20-min repeated interval LSPT, as a soccer-specific motor task, could induce a similar subjective MF as the 20-min Stroop task but with a better ecological validity. The novel task was designed to be intermittent, and it could represent the cognitive and soccer skill demands as real-life soccer play. Besides, the similar moderate intensity of the two motor tasks has excluded the interference of physical fatigue. The difference in cognitive demands accounts for the different efficiency of inducing MF. Apparently, the trait of this novel task was unexpected passing orders, which made it more cognitively demanding than the other motor task. The MF induced by the 20-min repeated interval LSPT and 20-min Stroop task had no similar effect on the cognitive and negative effect on soccer-specific skill performance. The findings supported the use of 20-min repeated interval LSPT as an MF-inducing task in soccer.

### Subjective Mental Fatigue

The 100-mm VAS was utilized by numerous studies as a primary method to evaluate subjective MF (Smith et al., 2015, 2016a,b,^2017^; Badin et al., 2016; Coutinho et al., 2017, 2018; Greco et al., 2017; Filipas et al., 2020; Kunrath et al., 2020b; Trecroci et al., 2020). In this study, the similar significant increases in subjective MF assessed by VAS after 10xLSPT and Stroop-20 and no significant changes after 10xC-LSPT indicated that the 20-min repeated interval LSPT could induce MF as well as the Stroop task.

The Stroop task needs important cognitive skills to achieve high performance as prolonged selective and sustained attention, as well as inhibitory control, which in turn causes a feeling of MF (Kunrath et al., 2020a). During LSPT, players need to perceive and interpret unpredictable passing orders and execute appropriate technical movements with the ball under time and space pressure (Ali et al., 2007). The test ideally includes perception-action couplings which represent the perceptual and information processing demands as in real-life gameplay (Pinder et al., 2011). Hence, the prolonged demand of decision-making and attention in 10xLSPT may have played a meaningful role in elevating the perception of MF. Besides, the specialty of LSPT made it more ecologically valid than the Stroop task in the MF inducement in the soccer context. Comparatively, the C-LSPT was only a repetition of a series of expected movements in a fixed order, which required a reduced cognitive demand, and therefore inefficient to induce significant elevation of perceived MF in this duration.

The subjective MF in this study, as the key indicator, was indicated by VAS. Despite its well-proven acceptable validity and reliability, VAS should be applied carefully as the primary measurement of MF in soccer research for several potential biases (Thompson et al., 2019). First, the subjective report was based on the understanding of MF of players. A poor understanding may lead to an over- or underestimation of the actual perception. While repeating the definition of MF might increase the difficulty of masking the purpose of the research and then increase the acquiescence bias, which means players tend to agree with the questions asked by investigators (Watson, 1992; Thompson et al., 2019). Moreover, the feeling of boredom and physical fatigue could interfere with the self-assessment of MF (Thompson et al., 2019). As mentioned earlier, Coutinho et al. (2017) adopted light general aerobic exercises for the control condition, but the MF condition was induced by a whole-body coordination task. The significantly higher intensity (i.e., HR) of this mentally fatiguing task might have caused an interference from physical fatigue in that study.

For minimizing the aforementioned bias, we customized the VAS, reduced the exposure times of the scale, adopted a stricter control of motor task (set 10xC-LSPT), and broke down the motor tasks into blocks (Thompson et al., 2019) by setting up intervals to prevent boredom and exhaustion. Players were blinded for the vernier readings, and the interval of pre-post reports was considered to be long enough (20 min). Combined with the similar intensity of two motor tasks (Figure 4), we concluded that the physical components had been controlled in this study. The extent of physical fatigue after two motor tasks did not account for the difference in the subjective MF of players.

### Cognitive Performance

It has been suggested that performance decrement during a cognitive task is the gold standard measure of MF (Hockey, 2011). Short versions of the Stroop task (50–62 stimuli, 2–3 min) have been validated and applied as tools for assessing MF by detecting the changes in response time and accuracy (Fortes et al., 2019, 2020; Gantois et al., 2020). In this study, we also adopted the 3-min Stroop task to assess MF. Our findings showed that the cognitive indicators remained steady after 10xLSPT and Stroop-20, while the response time after 10xC-LSPT showed a significant decrease with a moderate effect.

The unchanged outcome of response accuracy during three sessions supports the earlier study by Gantois et al. (2020). However, the response time was kept steady after the inducement of MF, which was not in line with previous findings (Fortes et al., 2019, 2020; Gantois et al., 2020). Although the reduction in cognitive performance is an indication of MF (Van Cutsem et al., 2017), it is suggested that cognitive performance does not necessarily need to worsen since compensatory effects may occur (i.e., an increase in brain activity and/or motivational component) (Van Cutsem et al., 2017). In fact, the high levels of motivation (approximately 87 of 100 AU) of players in this study may allow maintenance of cognitive performance under fatiguing conditions (van der Linden, 2011). Another plausible reason accounting for this discrepancy might be the slower baseline values of response time in this study (1.06 ± 0.2 s) than previous ones [0.9 ± 1.7 s (Fortes et al., 2019); 0.3 ± 0.2 s (Fortes et al., 2020); less than 0.6 s (Gantois et al., 2020)]. Although the participants in other abovementioned studies were at a professional level, high-level players tend to demonstrate better cognitive abilities than their low-level counterparts (Formenti et al., 2020; Athos et al., 2021), and the fastest response time in 10xC-LSPT (0.93 ± 0.15 s) indicated the possible underestimation of the response speed and potential slack of these well-trained collegiate players in 10xLSPT and Stroop-20.

### Soccer Skill Performance

The novel motor task (10xLSPT), similar to the 20-min continuous Stroop task, had a significant detrimental effect on technical ability and passing accuracy but no clear effect on movement time of LSPT. Where there were significant effects, the effect sizes were moderate to strong (technical ability: η^2^*_*p*_* = 0.608–0.640; passing accuracy: η^2^*_*p*_* = 0.385–0.482). These results of LSPT were in line with previous studies (Smith et al., 2016a,^2017^; Greco et al., 2017; Filipas et al., 2020).

Speed and accuracy are two fundamental aspects of skill performance in soccer. Due to a speed-accuracy trade-off (Lorist et al., 2000), studies showed that players tended to keep the speed at the expense of accuracy (Smith et al., 2016a). Based on the conceptual model developed by Smith et al. (2018) outlining a potential mechanism of MF, cognitively demanding tasks keep activating the anterior cingulate cortex, likely leading to elevated adenosine, and a corresponding decrease of dopamine in this brain region, which then causes impairments to many executive functions including attentional allocation (Smith et al., 2018). Mentally fatigued players would suffer from the limited amount of attention resources. Therefore, they may have found it easier to ensure the completion of LSPT than focus on the skill quality (Smith et al., 2016a; Kunrath et al., 2020a).

The 10xC-LSPT led to a positive effect on skill performance relative to the two mentally fatiguing tasks (10xLSPT and Stroop-20), and this could be interpreted as an increase in technique proficiency. The C-LSPT shared the same movement pattern as LSPT but with less mental demand. Repeating the sequences of movements in C-LSPT might promote the execution speed in posttest LSPT. There exists a close interplay of motor and cognitive skills in soccer players (Hans-Erik and Daniel, 2019). The attention window was positively correlated with dribbling skills, and working memory was positively associated with dribbling, control, and juggling skills (Athos et al., 2021). The series of movements in this predictable motor task might have the cognitive functions of activated players but without inducing cognitive fatigue, contributing to the enhancement of skill performance during the posttest. Logically, it is possible to utilize the motor task pattern as a supplementary training protocol for warm-up and re-warm-up in the future.

### Limitations and Future Directions

First, the participants in this study were collegiate players, whether the findings could be extended to professional players is unclear. Considering that professional players are believed to be more resistant to MF (Smith et al., 2018), further study could expand the task to a cohort of higher performance levels. Second, despite the strict control, the MF was primarily assessed with VAS which may have been influenced by the acquiescence bias (Watson, 1992). More objective psychophysiological measures of MF [e.g., electroencephalography (EEG)] are encouraged in the future (Thompson et al., 2019). Third, the cognitive performance test (Stroop-3) may be very easy for these collegiate players due to the lack of stricter time restrictions. A more sensitive and difficult cognitive performance test should be applied in the future.

## Conclusion

The 20-min repeated interval LSPT is able to induce a similar MF as the Stroop task but with a better ecological validity. The MF induced by the repeated interval LSPT had a similar impact as the Stroop task on cognitive and soccer-specific skill performance. The induced MF had detrimental effects on skill stability and passing accuracy. The novel motor task is recommended for studies in soccer when the inducement of MF is required. Practitioners should be cautious about the prolonged cognitive-demanding skill section of pre-match warm-up to avoid the negative effect of MF on the upcoming match. This motor task pattern could be followed as a supplementary training protocol.

## Data Availability Statement

The datasets presented in this study can be found in online repositories. The names of the repository/repositories and accession number(s) can be found in the article/supplementary material.

## Ethics Statement

The studies involving human participants were reviewed and approved by the Human Research Ethics Committee of Shanghai University of Sport. The patients/participants provided their written informed consent to participate in this study.

## Author Contributions

CB involved in the design of the study, data collection, analysis, and interpretation, as well as in the draft of the main document. YL worked on the data design, experiment organization, and manuscript content revision. AA and GN helped in the manuscript draft, design, data analysis, and revised them critically. All authors approved this final version and agreed to be accountable for all aspects of the work.

## Conflict of Interest

The authors declare that the research was conducted in the absence of any commercial or financial relationships that could be construed as a potential conflict of interest.

## Publisher’s Note

All claims expressed in this article are solely those of the authors and do not necessarily represent those of their affiliated organizations, or those of the publisher, the editors and the reviewers. Any product that may be evaluated in this article, or claim that may be made by its manufacturer, is not guaranteed or endorsed by the publisher.
